# Visualization and quantitation of fetal movements by real-time three-dimensional ultrasound with live xPlane imaging in the first trimester of pregnancy

**DOI:** 10.3325/cmj.2016.57.474

**Published:** 2016-10

**Authors:** Ye Lu, Taizhu Yang, Hong Luo, Feng Deng, Qianyun Cai, Weiwei Sun, Hao Song

**Affiliations:** 1 Department of Diagnostic Ultrasound, West China Second University Hospital, Sichuan University, Key Laboratory of Birth Defects and Related Diseases of Women and Children (Sichuan University), Ministry of Education, Chengdu, Sichuan, China; 2 Department of Obstetrics and Gynecology, West China Second University Hospital, Sichuan University, Key Laboratory of Birth Defects and Related Diseases of Women and Children (Sichuan University), Ministry of Education, Chengdu, Sichuan, China; 3 Department of Pediatrics, West China Second University Hospital, Sichuan University, Key Laboratory of Birth Defects and Related Diseases of Women and Children (Sichuan University), Ministry of Education, Chengdu, Sichuan, China; 4 Intensive Care Unit of Obstetrics and Gynecology, West China Second University Hospital, Sichuan University, Key Laboratory of Birth Defects and Related Diseases of Women and Children (Sichuan University), Ministry of Education, Chengdu, Sichuan, China

## Abstract

**Aim:**

To prove whether real-time three-dimensional (3D) ultrasound with live xPlane imaging is better in observing fetal movements than standard ultrasound imaging.

**Methods:**

50 healthy women with singleton pregnancies (22-43 years old) at 11 to 14 weeks of gestation underwent real-time 3D ultrasound examination with live xPlane imaging from July 2014 to February 2015. The incidence and frequency of 10 fetal movement patterns in 10 minutes were evaluated, including general movements (GMs), isolated arm movements, isolated leg movements, hiccup, stretching, breathing, startle, jaw opening, isolated head retroflexion, and isolated head anteflexion. The correlation between gestational age and frequency of each fetal movement pattern was analyzed.

**Results:**

GM had the highest incidence (100%), followed by startle (84%) and isolated arm movements (68%). Their median frequency was 5 (IQR 3-6), 5 (IQR 1.75-11.5), and 1 (IQR 0-2), respectively. GM (Z = 5.875, *P* < 0.001) and startle (Z = 5.302, *P* < 0.001) had significantly higher frequency than isolated arm movements. The other 7 fetal movement patterns had much lower incidence and frequency. The frequency of GM was positively correlated with gestational age (r = 0.360, *P* = 0.010).

**Conclusion:**

Real-time 3D ultrasound with live x Plane imaging was shown to be a feasible tool for observing fetal movements.

Fetal behavior is referred to as any observable fetal action, which may be observed by mother or recorded by real-time ultrasound (US) imaging. Fetal movements change with gestational age. There are rapid changes in position and posture at 9-12 weeks of gestation. In contrast, flexion and extension of the limbs, and longer episodes of changes in position occur at 13-16 weeks of gestation (1). An increasing number of studies have reported a discrepancy of fetal movement patterns between normally developing fetuses and the fetuses at risk (2,3). Also, assessment of fetal behavior could provide valuable data to differentiate normal brain development from abnormal brain development (4,5). Thus, fetal motility is increasingly considered a reflection of fetal neurodevelopment.

Fetal motility can be considered to be a spontaneous expression of the developing nervous system. The quantification of fetal movements has been shown to predict preterm birth and the absence of breathing movements served as a marker for preterm labor (6,7). The changes in the quality of general fetal movements identify the deterioration of the fetal central nervous system (8). Four-dimensional (4D) US technology has been applied to observe normal fetal facial expressions and fetal movements in all three trimesters (9). Nevertheless, it is limited by relatively low spatial resolution, staggering images, and a time-consuming procedure, which may affect the US biological effects and safety. The real-time three-dimensional (3D) processing facilitates simultaneous display of two high-resolution real-time images, thus acquiring a true midsagittal plane of the fetus (10,11). Real-time 3D echocardiography with live xPlane imaging has been used for scanning of the fetal heart (12). It is a simple and reliable method for assessment of fetal interventricular septum (13) and detection of fetal conotruncal anomalies (14). It has not been proved whether the real-time three-dimensional (3D) US with live xPlane imaging is a better method for observing fetal movements than US imaging.

In this study, real-time 3D US with live xPlane imaging was applied to observe and record the fetal movement patterns. Moreover, the incidence and frequency of each movement pattern was analyzed, and the correlation between frequency of each movement pattern and gestational age was evaluated. We attempted to prove the feasibility of the 3D US with live xPlane imaging in observing fetal movements.

## Patients and methods

### Patients

From July 2014 to February 2015, an observational study was conducted at the Department of Diagnostic Ultrasound and Department of Obstetrics and Gynecology of West China Women’s and Children’s Hospital. The study included 50 healthy pregnant women with normal singleton pregnancies who attended a routine US examination at 11 to 14 weeks of gestation. These women met the following inclusion criteria: no complications or clinical diseases; fetal crown-rump length (CRL) ranging from 45 to 84 mm; no abnormality detected by 2D US; normal fetal nuchal translucency (NT) measurement; delivery of neonates at term with normal 1- and 5-min Apgar scores (15). Exclusion criteria were pregnancies complicated by hypertensive disorders or congenital abnormalities; abnormal Apgar scores; preterm deliveries. The patients all offered signed informed consent before the study, which was approved by the ethics committee of West China Women’s and Children’s Hospital.

### Methods

All patients underwent real-time 3D US examination with live xPlane imaging, performed by one experienced operator (Y.L) using iU elite 3D US machine (Philips, Bothell, WA, USA) equipped with a trans-abdominal X6-1 (6 ~ 1 MHz) matrix-array transducer. According to a previous research (16), the examination was performed by using manufacturer’s NT preset, with mechanical index (MI) set at 0.4 and thermal index bone (TIB) set at 0.1. Following standard assessment in 2D B-mode US, the midsagittal view of a whole fetus was showed and zoomed as a region of interest, and the live xPlane function was activated. Subsequently, two real-time images of high-resolution were displayed simultaneously. The primary image plane was the midsagittal section of the whole fetus, displaying on the left part of the screen. The reference line was moved to the primary image line, and a secondary image plane cutting across the reference line was then exhibited on the right side of the screen. In the midsagittal section, the reference line was adjusted within the reference plane to be located along the fetal upper thorax. The axial section of the fetal upper thorax and arms (the secondary plane) was displayed in the right window. Because isolated arm movements might be missed in the midsagittal section, we chose axial section of the fetal upper thorax and arms as the secondary section. During the observation period, the probe was moved to both left and right parasagittal sections frequently, so as to avoid focusing on one section for a long time. The images displayed on the screen were recorded by videotape. The examination was performed for each participant in the same quiet room (temperature, 24-26°C).

According to the ALARA (As Low As Reasonably Achievable) principle (17), we determined the observation period to be 10 minutes. By analyzing the video recordings, we investigated 10 fetal movement patterns: GM, startle, stretching, isolated arm movements, isolated leg movements, hiccup, breathing, jaw opening, isolated head retroflexion, and isolated head anteflexion. Definitions of the 10 fetal movement patterns were in accordance with previous studies (5,18) (Table 1). Frequency and incidence of each fetal movement pattern during the observation period was evaluated by the same experienced observer.

**Table 1:** Definitions of fetal movement patterns analyzed in the study

Movement pattern	Definition
General movements	Series of movements with variable speed and amplitude, involve all parts of the body without distinctive patterning of body parts. Duration varies from a few seconds to about a minute
Startle	Quick generalized movements, starting in the limbs and spreading to the neck and trunk, only last about one second
Stretching	A complex motor pattern, always carried out at a slow speed and consists of the forceful extension of the back, retroflexion of the head, and external rotation and elevation of the arms
Isolated arm or leg movements	Rapid or slow movements, and may involve extension, flexion, external and internal rotation, or abduction and adduction of an extremity, without movements in other body parts
Hiccup	A hiccup consists of a jerky contraction of the diaphragm
Breathing	Fetal breathing movements are usually paradoxical in a way that every contraction of the diaphragm causes an inward movement of the thorax
Jaw opening	The opening may be either slow or quick
Isolated head retroflexion and anteflexion	Isolated head retroflexion and anteflexion of the head not associated with general movements. Usually carried out slowly, but they can also be fast and jerky

### Statistical analysis

Due to lack of controls, we did not perform the sample size analysis before the study. The SPSS statistical software (version 11.5) (SPSS Inc., Chicago, IL, USA) was used for calculations. Normality of data was tested by Kolmogorov-Smirnov test. Normally distributed data are expressed as mean ± standard deviation (SD), otherwise as median with interquartile range (IQR). Differences were analyzed using *t* test or Wilcoxon signed ranks test. The Spearman's coefficient was used to assess the correlation between gestational age and the frequency of fetal movements. *P* value <0.05 was considered to be significant.

## Results

### Participants

The mean maternal age of the eligible cases was 28.96 ± 4.15 (range 22-43) years, and the mean gestation age was 12.83 ± 0.82 (range 11-14) weeks. The mean NT and CRL values were 1.59 ± 0.38 (range 1.4-2.9) mm and 64.7 ± 10.6 (range 45-84) mm, respectively.

### Observation of 10 fetal movement patterns

**Figure 1:**
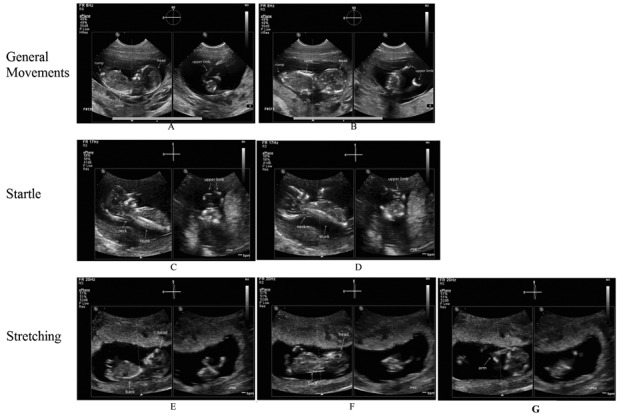
In-plane views of general movements, startle, and stretching by live xPlane imaging. (**A-B**) General movements involving the head, crump, spine, and limbs. (**C-D**) Startle. Quick generalized movement involving the neck, trunk, and limbs. (**E-G**) Stretching. Forceful extension of the back, retroflexion of the head, and elevation of the arms. The left side of each image is the primary image plane, while the right side is the secondary image plane (the axial section of the fetal upper thorax and arms). When the primary image is frozen, the original reference line disappears, thus the reference line in the primary image plane (left side) is added by the authors.

**Figure 2:**
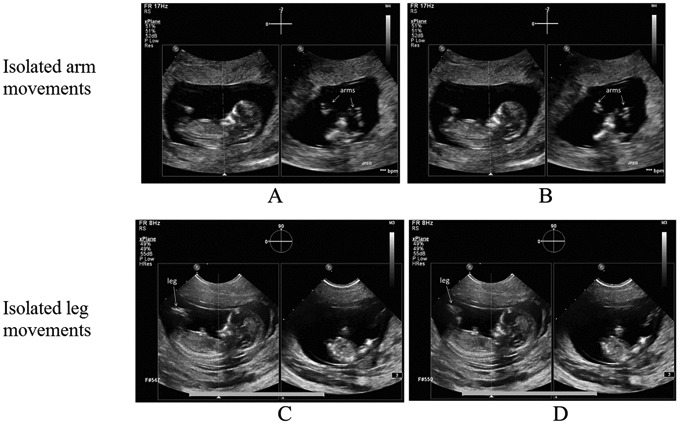
In-plane views of isolated arm movements and isolated leg movements by live xPlane imaging. (**A-B**) Movements of two arms are exhibited, without involvement of other body parts. (**C-D**) Isolated leg movements. Extension of one leg, without involvement of other body parts. The left side of each image is the primary image plane, while the right side is the secondary image plane (the axial section of the fetal upper thorax and arms). When the primary image is frozen, the original reference line disappears, thus the reference line in the primary image plane (left side) is added by the authors.

**Figure 3:**
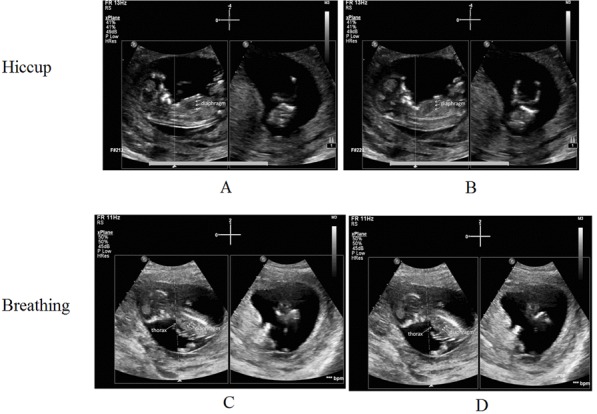
In-plane views of hiccup and breathing by live xPlane imaging. (**A-B**) Hiccup. A jerky movement of the diaphragm is seen. (**C**)-(**D**), Breathing. The movement of the diaphragm and the inward movement of the thorax. The left side of each image is the primary image plane, while the right side is the secondary image plane (the axial section of the fetal upper thorax and arms). When the primary image is frozen, the original reference line disappears, thus the reference line in the primary image plane (left side) is added by the authors.

**Figure 4:**
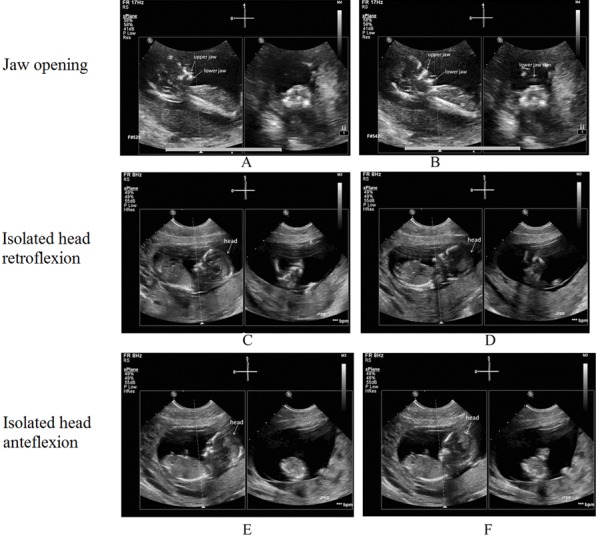
In-plane views of jaw opening, isolated head retroflexion, and isolated head anteflexion by live xPlane imaging. (**A-B**) The opening of upper and lower jaw. (**C-D**) Isolated head retroflexion. Movement of other parts of the body is not seen. (**E-F**) Isolated head anteflexion. Movement of other parts of the body is not seen. The left side of each image is the primary image plane, while the right side is the secondary image plane (the axial section of the fetal upper thorax and arms). When the primary image is frozen, the original reference line disappears, thus the reference line in the primary image plane (left side) is added by the authors.

GM (Figure 1 A-B), startle (Figure 1 C-D), stretching (Figure 1 E-G), isolated arm movements (Figure 2 A-B), isolated leg movements (Figure 2 C-D), hiccup (Figure 3 A-B), breathing (Figure 3 C-D), jaw opening (Figure 4 A-B), isolated head retroflexion (Figure 4 C-D), and isolated head anteflexion (Figure 4 E-F) were clearly observed by real-time 3D US with live xPlane imaging.

GM (general movements) was the most common movement pattern with 100% of incidence (Table 2). It was observed in all 50 fetuses and its median frequency was 5 (IQR 3-6, range 1-7) times. Startle was observed in 42 of 50 fetuses (84%) and its median frequency was 5 (IQR 1.75-11.5, range 1.75-11.5) times. Isolated arm movement was observed in 34 of 50 fetuses (68%) and its median frequency was 1 (IQR 0-2, range 0-3) time. GM (Z = 5.875, *P* < 0.001) and startle (Z = 5.302, *P* < 0.001) occurred significantly more often than isolated arm movements, whereas there was no significant difference in frequency between GM and startle. The other 7 fetal movement patterns (stretching, isolated leg movements, hiccup, breathing, jaw opening, isolated head retroflexion, and isolated head anteflexion) had a low incidence (10%-40%) and frequency (0), with varied frequency range during the observation period.

**Table 2:** Incidence and frequency of different types of fetal movement patterns*

Movement patterns	Fetuses (n)	Incidence (%)	Movement frequency range (times)	Median frequency (IQR)
General movements	50	100	1-7	5 (3-6) ^†^
Startle	42	84	0-34	5 (1.75-11.5) ^‡^
Stretching	20	40	0-5	0 (0-1)
Isolated arm movements	34	68	0-4	1 (0-2)
Isolated leg movements	10	20	0-4	0 (0-0)
Hiccup	11	22	0-2	0 (0-0)
Breathing	18	36	0-4	0 (0-0)
Jaw opening	9	18	0-3	0 (0-1)
Isolated head retroflexion	5	10	0-2	0 (0-0)
Isolated head anteflexion	7	14	0-5	0 (0-0)

*IQR – interquartile range.

†Z = 5.875, *P* < 0.001, comparing general movements with isolated arm movements. ‡Z = 5.302, *P* < 0.001, comparing startle with isolated arm movements.

### Correlation between the frequency of fetal movement and gestational age

**Figure 5:**
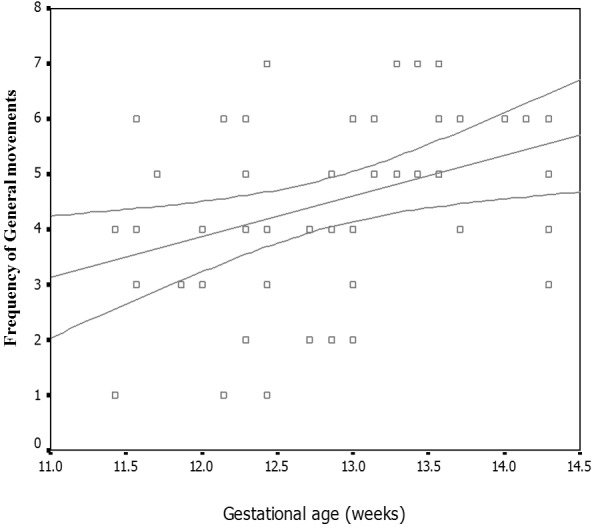
Scatter plot and linear regression analysis of frequency of general movement pattern vs the gestational age (weeks) (y = -4.913 + 0.732x; r = 0.360; *P* = 0.010). The frequency of GM was positively associated with gestational age (r = 0.360, *P* = 0.010).

Linear regression analysis was performed to evaluate the correlations between the frequency of each fetal movement pattern with gestational age (11-14 week). GM frequency showed significantly positive correlation with gestational age (r = 0.360, *P* = 0.010) (Figure 5). Frequency of the other 9 fetal movement patterns was not significantly correlated with gestational age.

## Discussion

In the present study, 10 fetal movement patterns were successfully detected by real-time 3D US with live xPlane imaging in 50 normal women with singleton pregnancies at 11-14 weeks of gestation. To our knowledge, this was the first time that real-time 3D US with live xPlane imaging was applied for observation and evaluation of fetal movements. Moreover, the study found that the frequency of GM was positively correlated with gestational age.

Evaluation of fetal movements could give useful insights into fetal functional characteristics and facilitate early detection of a range of prenatal neurological disorders and developmental dysfunctions (19,20). By using 4D US it is difficult to acquire a clear picture of specific movement patterns, such as hiccups, breathing, or jaw opening. Live xPlane imaging has been used to observe fetal heart chambers and aortic arches by providing high resolution view (21-23). Compared with conventional 2D US, real-time 3D US with live xPlane imaging adds another section and provides more information while the frame rate does not obviously decrease (24). With regard to the multiplanar view of 3D volumes acquired by conventional mechanical 3D probes, only the primary plane is acquired with full resolution while the other two planes are reconstructed. In contrast, live xPlane imaging displays two high-resolution real-time views of the target organ by using a matrix-array probe (11). In this research, the frame rate with live xPlane imaging was adjusted up to 19 Hz when zooming a whole fetus, enabling real-time visualization of fetuses in motion. The study suggests that it is a relatively simple and valid method for observation of fetal movements.

High-intensity focused US could effectively kill tumor cells and severely impair tumor blood vessels in human malignant carcinoma (25). Exposure to diagnostic US might lead to hyperthermia and tissue damage (26). Therefore, for fetal sonographic examinations safety issues are of utmost importance. Previous examinations of fetal movements using 4D US technology often took 15-30 minutes or longer (27-29). The present study determined the minimal exposure time and the lowest acoustic output that guaranteed adequate diagnostic acuity of live xPlane imaging based on the ALARA principle (17,30). The fetuses were observed for a relatively shorter period (10 minutes), and for the sake of safety the MI and TIB of live xPlane imaging was set at 0.4 and 0.1, respectively (31).

The incidence of GM and startle was 100% and 84%, respectively, which was similar to the results of Hantoushzadeh et al (90.8% and 74.2%, respectively) (32). Similarly, studies using 4D US have reported that GM is the most frequent fetal movement pattern (27,33,34). GMs emerge during early fetal life and are present until the end of the first half a year of life. They require the participation of all body parts, and are varied in intensity, force, and speed (35,36). Fetal onset of complex and variable GMs denotes the initiation of supraspinal modulation of spinal and brainstem circuitries. Perinatal assessment of GMs is a reflection of the integrity of nervous system (37). Fetal and infant GMs might provide useful information for predicting neurodevelopmental disabilities (38). This study found that of the 10 fetal movement patterns, GM was the only one movement pattern whose frequency was positively related to gestational age (11-14 week). Another study also reported that frequency of fetal movement patterns tends to increase with increased gestational age. Our result indicates that GMs might be used as a primary parameter for evaluation of fetal movements during 11-14 weeks of gestation. More studies are needed to disentangle the relationship between frequency of GMs and gestational age.

The limitation of this study is that it did not have a control group of patients (with abnormalities), nor did it have a control group with which it could be compared. The two sections obtained by real-time 3D US with live xPlane imaging could not include every part of the fetal body. Therefore, subtle fetal movements might not have been visualized in the midsagittal section or axial section of the fetal upper thorax and arms. This study only observed fetal movement during 11-14-week gestational period. Further studies should attempt to assess fetal movements in all three trimesters. Comparative studies with other alternative methods for observation of fetal movements are also required to yield more evidence supportive of application of live xPlane imaging. Additionally, fetal movements were analyzed by one sonologist and intra-observer agreement was not analyzed. The results obtained in this study should be validated by further studies including more sonologists with an acceptable degree of inter-observer agreement.

The study suggests that real-time 3D US with live xPlane imaging is a feasible tool for observation of fetal movements, compared with 4D US. GMs might be recommended as a promising variable for assessing the fetal movements at 11-14 weeks of gestation. Further studies are needed to verify the reproducibility and sensitivity of real-time 3D US with live xPlane imaging for perceiving fetal movements.

## References

[1] Reinold E (1979). Identification and differentiation of fetal movements.. Contrib Gynecol Obstet.

[2] Birnholz JC, Stephens JC, Faria M (1978). Fetal movement patterns: a possible means of defining neurologic developmental milestones in utero.. AJR Am J Roentgenol.

[3] De Vries J, Fong B (2007). Changes in fetal motility as a result of congenital disorders: an overview.. Ultrasound Obstet Gynecol.

[4] Kurjak A, Tikvica A, Stanojevic M, Miskovic B, Ahmed B, Azumendi G (2008). The assessment of fetal neurobehavior by three-dimensional and four-dimensional ultrasound.. J Matern Fetal Neonatal Med.

[5] Vries JD, Visser G, Prechtl H (1985). The emergence of fetal behaviour. II. Quantitative aspects.. Early Hum Dev.

[6] Besinger RE, Compton AA, Hayashi RH (1987). The presence or absence of fetal breathing movements as a predictor of outcome in preterm labor.. Am J Obstet Gynecol.

[7] Castle BM, Turnbull AC (1983). The presence or absence of fetal breathing movements predicts the outcome of preterm labour.. Lancet.

[8] Sival DA, Visser GH, Hf P (1992). The effect of intrauterine growth retardation on the quality of general movements in the human fetus.. Early Hum Dev.

[9] Kurjak A, Stanojevic M, Andonotopo W, Scazzocchio-Duenas E, Azumendi G, Carrera JM (2005). Fetal behavior assessed in all three trimesters of normal pregnancy by four-dimensional ultrasonography.. Croat Med J.

[10] Hata T, Dai SY, Marumo G (2010). Ultrasound for evaluation of fetal neurobehavioural development: from 2-D to 4-D ultrasound.. Infant Child Dev.

[11] Xiong Y, Wah Y, Chan L, Leung T, Fung T, Lau T (2010). Real-time three-dimensional ultrasound with Live xPlane imaging assists first-trimester acquisition of a true midsagittal section.. Ultrasound Obstet Gynecol.

[12] Xiong Y, Che M, Lin WC, Ting YH, Fung TY, Leung TY (2012). Scan the fetal heart by real-time three-dimensional echocardiography with live xPlane imaging. J Matern Fetal Neonatal Med.

[13] Xiong Y, Wah Y, Chen M, Leung T, Lau T (2009). Real-time three-dimensional echocardiography using a matrix probe with live xPlane imaging of the interventricular septum.. Ultrasound Obstet Gynecol.

[14] Xiong Y, Liu T, Gan HJ, Wu Y, Xu JF, Ting YH (2013). Detection of the fetal conotruncal anomalies using real-time three-dimensional echocardiography with live xPlane imaging of the fetal ductal arch view.. Prenat Diagn.

[15] Nelson KB, Ellenberg JH (1981). Apgar scores as predictors of chronic neurologic disability.. Pediatrics.

[16] Einspieler C, Prayer D, Prechtl HF. Fetal behaviour: a neurodevelopmental approach: Mac Keith; 2012.

[17] Sasaki M, Yanagihara T, Naitoh N, Hata T (2010). Four-dimensional sonographic assessment of inter-twin contact late in the first trimester.. Int J Gynaecol Obstet.

[18] Kurjak A, Andonotopo A, Hafner W, Stanojevic A, Azumendi M, Ahmed G (2006). Normal standards for fetal neurobehavioral developments–longitudinal quantification by four-dimensional sonography.. J Perinat Med.

[19] Abo-Yaqoub S, Kurjak A, Mohammed AB, Shadad A, Abdel-Maaboud M (2012). The role of 4-D ultrasonography in prenatal assessment of fetal neurobehaviour and prediction of neurological outcome.. J Matern Fetal Neonatal Med.

[20] Vermeulen R, Peeters-Scholte C, Van Vugt J, Barkhof F, Rizzu P, van der Schoor SR (2011). Fetal origin of brain damage in 2 infants with a col4a1 mutation: Fetal and neonatal MRI. Neuropediatrics.

[21] Yuan Y, Leung K, Ouyang Y, Yang F, Tang M, Chau A (2011). Simultaneous real-time imaging of four-chamber and left ventricular outflow tract views using xPlane imaging capability of a matrix array probe.. Ultrasound Obstet Gynecol.

[22] Xiong Y, Chen M, Chan LW, Ting YH, Fung TY, Leung TY (2012). Scan the fetal heart by real-time three-dimensional echocardiography with live xPlane imaging.. J Matern Fetal Neonatal Med.

[23] Xiong Y, Chen M, Chan L, Ting Y, Fung T, Leung T (2012). A novel way of visualizing the ductal and aortic arches by real-time three-dimensional ultrasound with live xPlane imaging.. Ultrasound Obstet Gynecol.

[24] Roberson DA, Cui VW (2011). Evaluation of Atrial and Ventricular Septal Defects with Real-Time Three-Dimensional Echocardiography: Current Status and Literature Review.. Curr Cardiovasc Imaging Rep.

[25] Wu F, Chen WZ, Bai J, Zou JZ, Wang ZL, Zhu H (2001). Pathological changes in human malignant carcinoma treated with high-intensity focused ultrasound.. Ultrasound Med Biol.

[26] O’Brien WD, Deng CX, Harris GR, Herman BA, Merritt CR, Naren S (2008). The risk of exposure to diagnostic ultrasound in postnatal subjects: thermal effects.. Journal of Ultrasound in Medicine..

[27] Kurjak A, Stanojevic M, Andonotopo W, Scazzocchio-Duenas E, Azumendi G, Jm C (2005). Fetal behavior assessed in all three trimesters of normal pregnancy by four-dimensional ultrasonography.. Croat Med J.

[28] Yigiter AB, Kavak ZN (2006). Normal standards of fetal behavior assessed by four-dimensional sonography.. J Matern Fetal Neonatal Med.

[29] Andonotopo W, Medic M, Salihagic Kadic A, Milenkovic D, Maiz N, Scazzocchio E (2005). The assessment of fetal behavior in early pregnancy: comparison between 2D and 4D sonographic scanning.. J Perinat Med.

[30] Don S (2004). Radiosensitivity of children: potential for overexposure in CR and DR and magnitude of doses in ordinary radiographic examinations.. Pediatr Radiol.

[31] Einspieler C, Prechtl HF (2005). Prechtl’s assessment of general movements: a diagnostic tool for the functional assessment of the young nervous system.. Ment Retard Dev Disabil Res Rev.

[32] Hantoushzadeh S, Sheikh M, Shariat M, Farahani Z (2015). Maternal perception of fetal movement type: the effect of gestational age and maternal factors. The Journal of Maternal-Fetal and Neonatal Medicine.

[33] Lüchinger AB, Hadders-Algra M, Van Kan CM, de Vries JI (2008). Fetal onset of general movements.. Pediatr Res.

[34] De Vries J, Visser G, Prechtl HF (1982). The emergence of fetal behaviour. I. Qualitative aspects.. Early Hum Dev.

[35] Einspieler C, Prechtl HFR (2005). Prechtl’s assessment of general movements: A diagnostic tool for the functional assessment of the young nervous system.. Ment Retard Dev Disabil Res Rev.

[36] Nelson TR, Fowlkes JB, Abramowicz JS, Church CC (2009). Ultrasound biosafety considerations for the practicing sonographer and sonologist.. J Ultrasound Med.

[37] Lüchinger AB, Mijna HA, Kan CM (2008). Van, Vries JIP, De. Fetal onset of general movements.. Pediatr Res.

[38] Zuk L (2011). Fetal and infant spontaneous general movements as predictors of developmental disabilities.. Dev Disabil Res Rev.

