# Correction: Lu et al: Visualization and quantitation of fetal movements by real-time three-dimensional ultrasound with live xplane imaging in the first trimester of pregnancy

**DOI:** 10.3325/cmj.2017.58.84

**Published:** 2017-02

This corrects the article “Visualization and quantitation of fetal movements by real-time three-dimensional ultrasound with live xplane imaging in the first trimester of pregnancy” in volume 57 on page 474.

In the article by Lu Y et al “Visualization and quantitation of fetal movements by real-time three-dimensional ultrasound with live xplane imaging in the first trimester of pregnancy”, published in the Croatian Medical Journal (1), the name of the first author was incorrectly written as Ye Lu instead of Lu Ye, which causes problems with retrieval of articles co-authored by Lu Ye from bibliographical databases. The correct information on the article should be as follows: Lu Y, Yang T, Luo H, Deng F, Cai Q, Sun W, Song H. Visualization and quantitation of fetal movements by real-time three-dimensional ultrasound with live xplane imaging in the first trimester of pregnancy. Croat Med J, 2016,57:474-81. On page 476, in the second paragraph of the Results section, the ‘range 1.75-11.5′ should be revised to ‘range 0-34’, and ‘range 0-3′ should be revised to ‘range 0-4’. Figure 3 was incorrect and should be replaced with the following correct figure (Figure 1). All changes have already been included in the pdf version available on the Croatian Medical Journal website.

**Figure 1:**
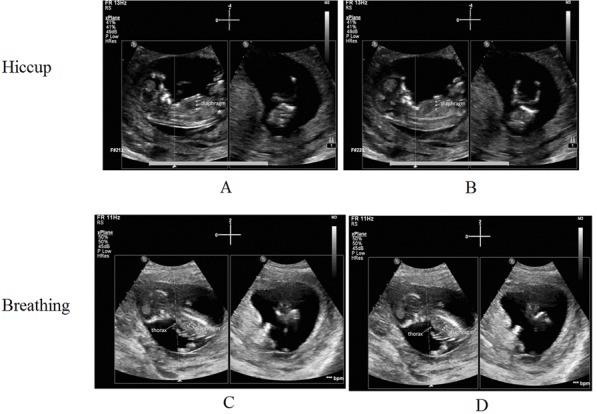
In-plane views of hiccup and breathing by live xPlane imaging. (A-B) Hiccup. A jerky movement of the diaphragm is seen. (**C**)-(**D**), Breathing. The movement of the diaphragm and the inward movement of the thorax. The left side of each image is the primary image plane, while the right side is the secondary image plane (the axial section of the fetal upper thorax and arms). When the primary image is frozen, the original reference line disappears, thus the reference line in the primary image plane (left side) is added by the authors.
